# Advancements in magnetic nanoparticle-based biosensors for point-of-care testing

**DOI:** 10.3389/fbioe.2024.1393789

**Published:** 2024-04-25

**Authors:** Miaomiao Wang, Lian Jin, Polly Hang-Mei Leung, Franklin Wang-Ngai Chow, Xiaoni Zhao, Hui Chen, Wenjing Pan, Hongna Liu, Song Li

**Affiliations:** ^1^ Hunan Key Laboratory of Biomedical Nanomaterials and Devices, Hunan University of Technology, Zhuzhou, China; ^2^ Department of Health Technology and Informatics, The Hong Kong Polytechnic University, Hong Kong SAR, China; ^3^ Guangzhou Wanfu Biotechnology Company, Guangzhou, China; ^4^ Hengyang Medical School, University of South China, Hengyang, China; ^5^ National Health Commission Key Laboratory of Birth Defect Research and Prevention, Hunan Provincial Maternal and Child Healthcare Hospital, Changsha, China; ^6^ Key Laboratory of Rare Pediatric Diseases, Ministry of Education, University of South China, Hengyang, China

**Keywords:** magnetic nanoparticles, POCT, biosensors, biological detection, separation

## Abstract

The significance of point-of-care testing (POCT) in early clinical diagnosis and personalized patient care is increasingly recognized as a crucial tool in reducing disease outbreaks and improving patient survival rates. Within the realm of POCT, biosensors utilizing magnetic nanoparticles (MNPs) have emerged as a subject of substantial interest. This review aims to provide a comprehensive evaluation of the current landscape of POCT, emphasizing its growing significance within clinical practice. Subsequently, the current status of the combination of MNPs in the Biological detection has been presented. Furthermore, it delves into the specific domain of MNP-based biosensors, assessing their potential impact on POCT. By combining existing research and spotlighting pivotal discoveries, this review enhances our comprehension of the advancements and promising prospects offered by MNP-based biosensors in the context of POCT. It seeks to facilitate informed decision-making among healthcare professionals and researchers while also promoting further exploration in this promising field of study.

## 1 Introduction

Point-of-care testing (POCT) is a vital component of modern healthcare by providing rapid diagnostic results at the POC, ultimately enhancing patient care standards (Islam et al., 2020). It is an innovative immunodiagnostic technique that offers real-time clinical diagnosis, distinguished by its exceptional sensitivity and rapidity, and is experiencing a rapid growth in the industry (Luppa et al., 2011). In modern healthcare, POC testing is a vital component, particularly in the diagnosis of specific diseases (Parihar et al., 2023c). For instance, in acute myocardial infarction (AMI) diagnosis, timely and accurate identification is crucial for reducing patient mortality rates. POC testing enables rapid detection of changes in biomarker levels, such as cardiac troponin I (cTnI), in the blood. This aids physicians in promptly determining whether a patient has experienced myocardial infarction (Boeddinghaus et al., 2020). Similarly, in diabetes management, monitoring blood glucose levels essential. POC testing allows for the quick measurement of patients’ blood glucose levels, enabling physicians and patients to adjust medications and diet effectively to maintain blood glucose levels within the appropriate range (Gourlay et al., 2024). POCT encompasses a wide range of applications, including clinical laboratories (Van Hoof et al., 2022; Xiao et al., 2022), chronic disease management (Herbin et al., 2020; Bots et al., 2022), drug monitoring (Young et al., 2019; Ates et al., 2022; Wang X. et al., 2022; Chen Z. et al., 2022; Song et al., 2022; Beduk et al., 2023), food safety assessment (Tang et al., 2019; Hu et al., 2021; Zaczek-Moczydlowska et al., 2021), environmental quality testing (Xu et al., 2020), and emergency response measures, setting it apart from traditional testing methods.

In contrast to conventional testing approaches that are labor-intensive, time-consuming, and reliant on large instruments like Chemiluminescence Analyzers (Sun et al., 2020; Cacicedo et al., 2023), POCT offer several advantages. These include swift turnaround times, minimal analysis duration, and a user-friendly operating procedure. As a result, POCT expedites disease diagnosis, improves technician efficiency, and saves valuable time for both patients and physicians (Zhang L. et al., 2022). This transformative shift in global medical hygiene holds the potential to revolutionize healthcare practices worldwide.

Traditional diagnostic methods, such as polymerase chain reaction (PCR), immunofluorescence technique (FIA), immune colloidal gold technique (CGIA), and chemiluminescence immunoassay (CLIA), often require expensive equipment and lengthy experimental procedures (Shen et al., 2014). Emerging detection systems that offer ease of operation and rapid analytical results stand to benefit greatly from POCT. In clinical testing, certain metabolites such as CO_2_, urea, inorganic salts, and excess water can influence the sensitivity and selectivity of PCR reactions (Wen et al., 2014). These effects may involve changes in the activity and stability of DNA polymerase, alterations in the pH and ion concentration of the reaction buffer, and interference with the primer-template DNA pairing. Such factors can reduce the efficiency of PCR reactions, leading to potential false positives or negatives. Hence, POCT instruments with rapid detection capabilities and sensitive biosensors offer substantial advantages in clinical settings.

Biosensors traditionally consist of biological recognition elements (receptors), signal transducers (conversion transducers), and detectors (Capriotti et al., 2019). Magnetic nanoparticles (MNPs) constitute a unique class of nanoparticles (NPs) that significantly enhance biosensor efficiency and selectivity (Wang Y. et al., 2022). They possess properties such as high biomolecule binding rates (Li B. et al., 2017), large specific surface areas (Kellnberger et al., 2016), low toxicity (Lee et al., 2016), and magnetic enrichment capabilities (Su et al., 2019; Gavilán et al., 2021), making them an excellent choice for various biological assays. Moreover, MNPs can be tailored to different analyses (Ren et al., 2020), reducing both cost and time (Lin et al., 2017; Yang et al., 2022). Additionally, they are environmentally friendly and pose no harm to human health.

For instance, Li J. et al. (2017) developed a magnetic nano-biosensor utilizing MNPs, fluorescence-activated cell sorting (FACS), and magnetic separation for the rapid clinical diagnosis of cancer patients, particularly in detecting circulating tumor cells (CTCs). MNPs also find applications in diverse biomedical fields, including targeted drug delivery (Guo et al., 2022), bioimaging (Zong et al., 2021), targeted hyperthermia for cancer treatment (Zhao et al., 2019; Dash et al., 2022), as well as the capture, isolation, concentration, and detection of nucleic acids (Zhao et al., 2023), bacteria (Behzad et al., 2022), and viruses (Labib et al., 2021; Xing et al., 2022), They have proven effective in immunoassays and immune sensors (Ha and Kim, 2022; Li et al., 2023).

In recent years, there has been significant progress in synthesizing and applying MNPs, with numerous studies discussing various synthesis methods (Adewunmi et al., 2021; Billings et al., 2021; Shukla et al., 2021; Niculescu et al., 2022; Parihar and Khan, 2023). The continuous advancement of science and technology has enriched and improved the preparation methods of MNPs, leading to their widespread use in fields such as biomedicine, environmental science, and materials science. Researchers are dedicated to enhancing the efficiency, purity and stability of MNPs by exploring different synthesis routes and optimizing preparation conditions to meet the demands for high-performance MNPs in various fields. Meanwhile, the dynamic and promising nature of MNPs is attributed to their surface modification capabilities, facilitating interactions with target substances and the isolation of specific molecules from complex biological environments. MNPs can also amplify detection signals, thanks to their high specific surface areas, thereby increasing biosensor sensitivity (Parihar et al., 2023b). Moreover, MNPs enable POCT through various methods, including optical, magnetic, and electrochemical approaches, as well as combined detection devices and microfluidic technology. This makes them a critical component in achieving rapid, convenient, and cost-effective clinical diagnoses (Sivakumar and Lee, 2022).

In summary, this review underscores the pivotal role of MNPs in transforming POCT. Their versatility, sensitivity, and multifaceted applications make them indispensable in clinical diagnoses. This comprehensive review covers a wide range of MNPs functions and applications in bioassays, exploring the latest advancements in MNPs-based biosensors, with a specific focus on their integration into POCT.

## 2 The impact of magnetic nanoparticles on biological detection

Sample preparation is crucial for detecting qualitative and quantitative trace analytes in complex biological samples (Chen et al., 2021). Factors that can affect the detection of analytes in a sample include concentration, separation, enrichment, and derivatization. In essence, sample preparation refers to the process of eliminating interfering substances from biological samples, thereby reducing their inherent complexity (Wang et al., 2023). The primary goal is to enhance the accuracy, sensitivity, and selectivity of subsequent analytical measurements and analyses by selectively removing or minimizing interferences, such as matrix components, contaminants, or co-existing analytes. The most common biological samples, or liquids such as urine, plasma, serum, and saliva. Typical techniques for sample separation include protein precipitation and centrifugation. The general phases of sample extraction comprise of cell or tissue disruption. removal of membrane lipids and proteins, and other contaminants by denaturing and inactivating nucleic acids, and the subsequent concentration and purification of nucleic acids (Tavallaie et al., 2018).

Due to the high surface area and distinctive physicochemical features of MNPs, nanomaterials have been developed rapid and effective sample analysis, thanks to the rapid growth of nanotechnology. These materials enable the magnetic separation of biological targets from the original sample without damaging the biological samples and reducing unspecific absorption of interfering biomolecules (Meng et al., 2023). This is critical for the development of real-time assay systems, providing a more convenient platform for extracting desired analytes from patient samples. With its advantages of precision, speed, mobility, simplicity, and reduced cost, this technology streamlines the process, produces easier-to-read data, and enhances clinical decision-making efficiency.

Extensive research has been dedicated to studying MNPs due to their two intrinsic characteristics: superparamagnetism and a high surface area-to-volume ratio. Superparamagnetic nanoparticles are nano-sized particles with magnetic responsiveness, typically having a diameter of less than 30 nm. MNPs exhibit a superparamagnetic state when the particle size falls below the critical size for superparamagnetic effects. Within this size range, MNPs not only remain unaggregated in the absence of external magnets but also lack a net magnetization strength (Hou et al., 2022). As a result, they can rapidly disperse in liquids, facilitating the efficient identification of bacteria, cells, biomolecules and proteins. Simultaneously, their fast diffusion rate and highly specific surface area contribute to the enrichment and effectiveness of ions, inorganic compounds, and organic compounds. Moreover, the properties of superparamagnetism and high surface-to-volume ratio are closely related to the shape, size, strength, and surface chemistry of magnetic nanoparticles, making them highly adaptable for magnetic biosensor applications. Herein, we summarized several representative magnetic nanomaterials for Biological detection (Table 1).

**TABLE 1:** Representative Magnetic nanomaterials for Biological detection.

Biomedical materials	Experimental sample	Combination	Advantages	References
Ab-MNPs	*Staphylococcus aureus*	Portable digital fluorescence reader	High sensitivity and high specificity	Kaushal et al. (2022)
MNP	Low abundance cell	Assisted microfluidic system	High recovery efficiency	Sun et al. (2022)
MNPs	*S. typhimurium*	Probe sandwich complexes	\	Liu et al. (2022)
MNPs	AMI.	ICTS	Highly sensitive, quantitative and dual-readout	Gong et al. (2018)
Fe_3_O_4_@SiO_2_	sEVs	MCAs-based ELISA	High stability	Yang et al. (2021)
MNPs	Pathogenic bacteria	Cationic polymer chains	Rapid capture and enrichment	Kim et al. (2021)
MNPs	Monoclonal antibodies	VIM polyme	Rapid and high efficiency separation	Zhou et al. (2023)
Fe_2_O_3_	respiratory viruses	Magneto-opto-fluidic (iMOF)	Rapid analysis of multiple swine respiratory viruses	Zhang Q. et al. (2022)
MNPs	*Salmonella* enteritidis	Amp	Cost-effective, non labor intensive, stable, sensitive and efficient	Bu et al. (2020)
Magnetic quantum dot	SARS-CoV-2	LFIA	High accuracy, specificity, and stability in saliva	Wang et al. (2021)
AuNPs	protein biomarkers	pNIPAAm	Rapid capture and enrichment	Nash et al. (2010)

### 2.1 Sample extraction detection and separation

**FIGURE 1:**
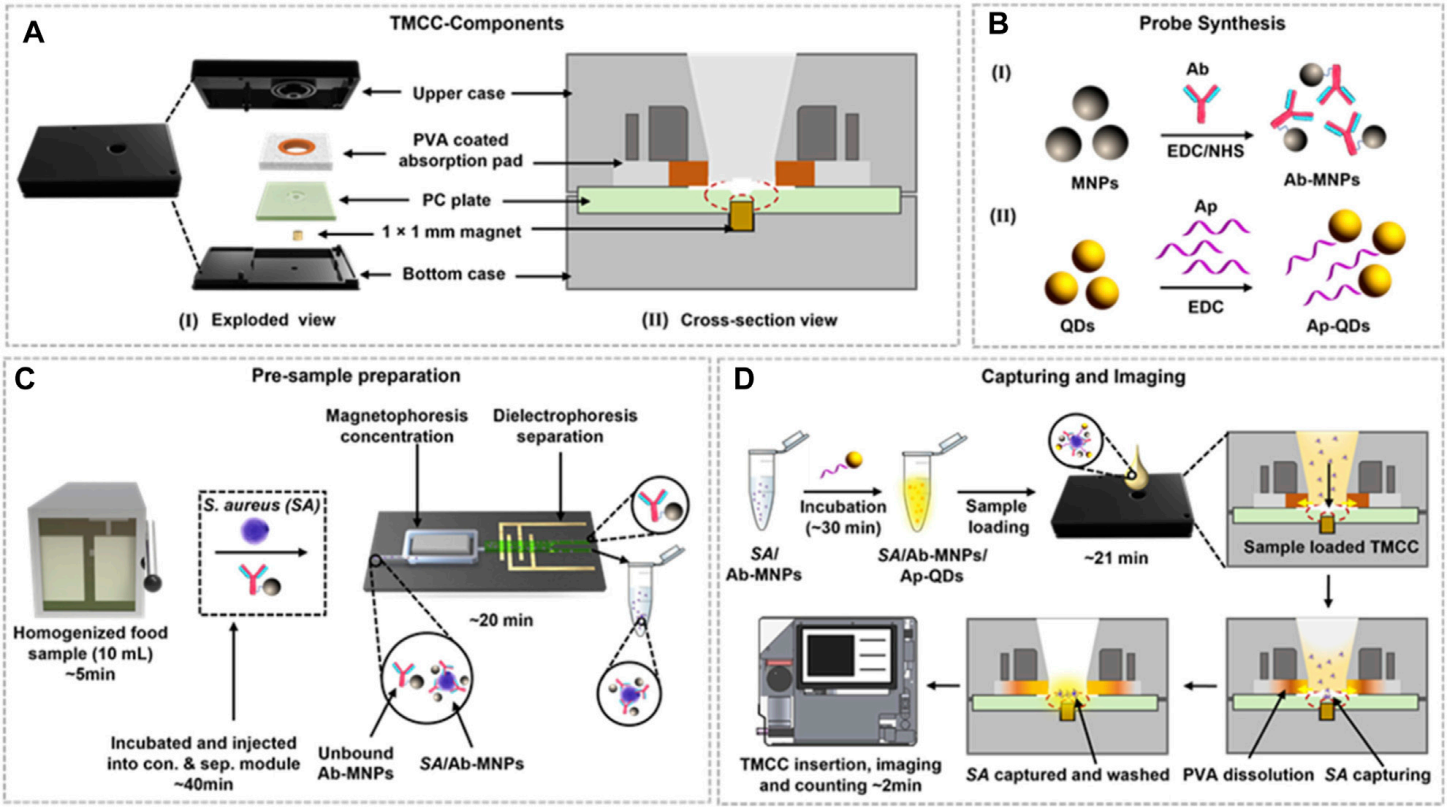
Schematic representation of the TMCC design, bacterial capture, and quantification process. **(A)** Exploded view (I) and cross-sectional (II) view of the TMCC and its sequential assembly. **(B)** Development of Ab-conjugated MNPs (Ab-MNPs) as the capture probe (I) and aptamer-conjugated QDs (Ap-QDs) as the imaging probe (II) using carbodiimide coupling. **(C)** Pre-sample preparation of the homogenized food sample spiked with *S. aureus* cells and incubation with the capture probe (Ab-MNPs) to concentrate the sample and separate unbound probes and food matrices. **(D)** Loading of recovered *S. aureus* cells bound with the detection probe into the TMCC for bacterial capture, followed by washing to remove unbound Ap-QD detection probes. The quantification process is conducted using a portable fluorescence reader based on digital imaging. Reprinted with permission from Kausha et al. (2019).

The separation of biological samples is a crucial step in their subsequent. Ideal separation methods should be simple to operate, offer high resolution, specificity, and short analysis times. MNPs play a pivotal role in achieving successful separation of biological samples due to their unique magnetic properties. MNPs enable specific identification, concentration, and contamination-free separation of samples, facilitating rapid solid-liquid separation when subjected to an applied magnetic field. This feature makes automated operations feasible. When a magnetic field is applied, MNPs aggregate toward the magnet, and upon removing the magnetic field, MNPs disperse in solution. Consequently, MNPs have found extensive use in the separation and purification of various biological samples, including cells (Bhati et al., 2021), proteins (Sharma et al., 2022), and nucleic acids (Nguyen et al., 2022; Huang et al., 2023). MNPs are particularly effective in recognizing whole-cell bacteria in large samples without the need for enrichment processes, concentration, identification, or quantification of bacterial samples. For example, in the concentration of bacteria in large samples, Kaushal et al. (Kaushal et al., 2022) developed a tunable magnetic capture box (TMCC) that combines *Staphylococcus* aureus-specific antibodies with magnetic nanoparticles in controlled liquid samples for magnetophoretic concentration and capture (Figure 1). The size of the MNPs and the strength of the magnetic field determine the duration required to capture the bacteria.

Quantification is carried out using digital images captured by a portable reader to observe the reaction of the target probe with bacteria and human cells. MNPs exhibit rapid responses and swift movement in the presence of an external magnetic field. Importantly, the identification and separation of target substances, enabling effective separation and purification (Tang et al., 2020). In contrast to traditional biological separation methods, which can potentially cause damage to biological samples, magnetic separation methods offer simplicity. They do not require high-performance liquid chromatography (HPLC) systems or filtration and centrifugation steps (Zhang Y. et al., 2022). The analysis procedure involving MNPs is straightforward, easy to operate, and portable device-based signal reading is convenient, making it suitable for POCT in biomedical diagnostics, benefiting human health.

**FIGURE 2:**
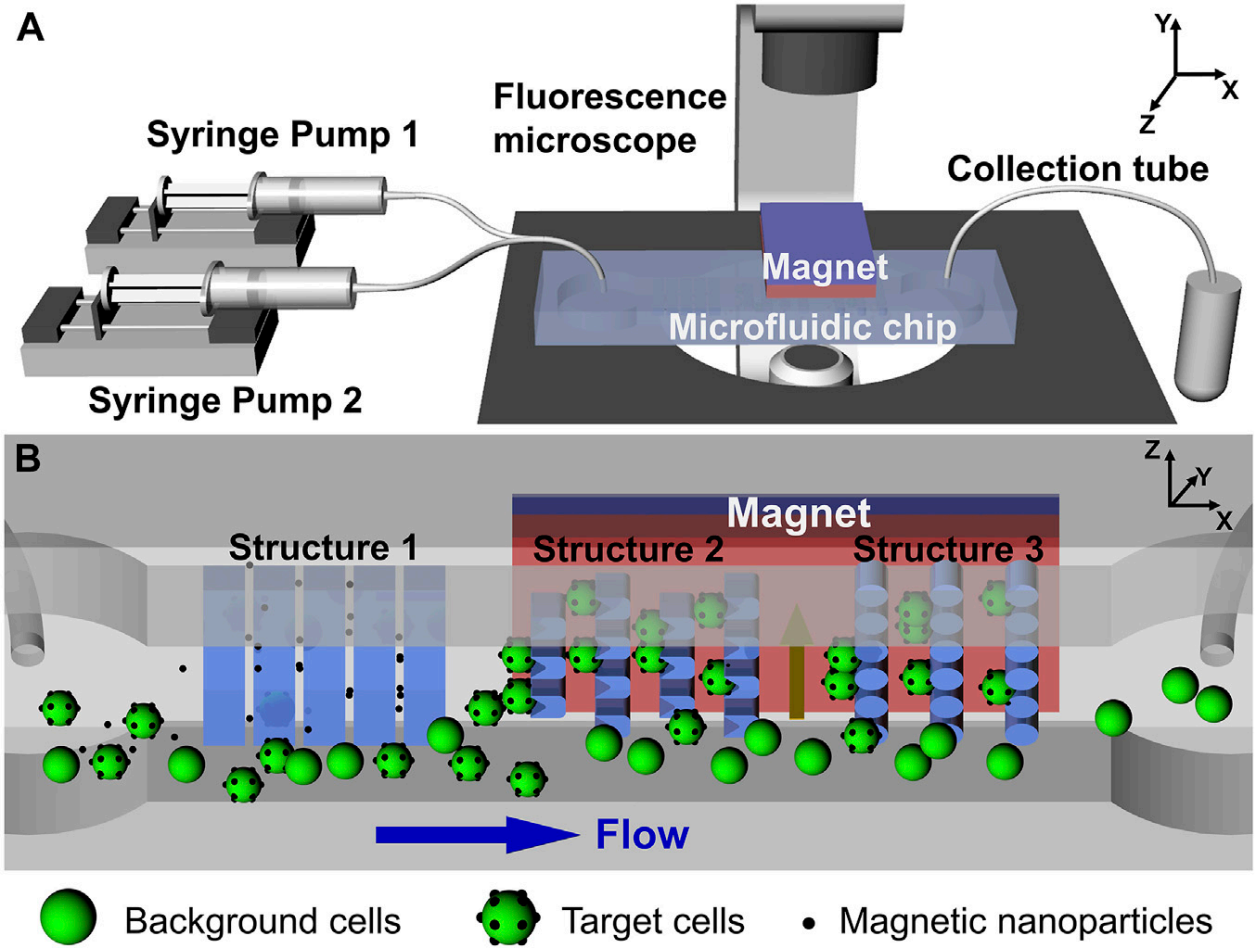
**(A)** Schematic representation of the active-passive integrated microfluidic system. **(B)** Internal view of the microfluidic chip depicting three types of microstructures and the applied magnetic field, illustrating the passive and active capture mechanisms, respectively. Reprinted with permission from Sun Y. et al. (2022).

Moreover, the integration of MNPs and microfluidics enhances the performance of bioanalytical systems by incorporation functionalized magnetic nanoparticles into microchip devices (Zhong et al., 2023) Microfluidic chips, which incorporate mixing, separation, washing, and detection functions, are commonly used for the automated detection of pathogenic bacteria. For instance, Sun T. et al. (2022) developed a microfluidic system that utilizes MNPs labeling to tag target cells and employs magnetic fields for separation, enabling the effective isolation of low-abundance cells (Figure 2).

This technology has also paved the way for liquid biopsies and fundamental biomedical research, offering excellent target cell recovery and successful isolation of low-abundance Hela cells. Notably, Liu et al. (2022) introduced a novel approach. They simultaneously injected bacterial samples into a microfluidic chip using capture antibody-modified MNPs and enzyme probes for detecting antibody and glucose oxidase (Gox) modifications. These components were mixed and incubated to form MNPs bacterial-probe sandwich complexes. Subsequently, under the influence of an applied magnetic fields, these complexes were magnetically captured in the separation chamber. Finally, high-impedance glucose was introduced into the isolation chamber to determine the amount of the complex, enabling rapid and sensitive detection of *Salmonella*.

### 2.2 Markers and carriers for biomolecules

The detection and manipulation of biomolecules on a versatile platform hold significant value not only in devices like diagnostic tools but also in the fundamental research of biological and medical systems (Bhardwaj et al., 2019; Clifford et al., 2021; Hwang et al., 2021; Chen Z. et al., 2023). In recent years, there has been a proliferation of electrochemical biomarker sensors employing various biomarkers, such as biomaterials (Mathew et al., 2021), two-dimensional materials (Bakirhan et al., 2018), and fluorescent materials (Wang et al., 2017). Biomarkers like proteins, exosomes, nucleic acids, and viruses play pivotal roles in identifying structural or operational changes in systems, organs, tissues, cells, or subcells. Currently, several methods allow for specific quantitative detection of biomarkers, including radioimmunoassay (Kaplan et al., 1981; Grange et al., 2014), enzyme-linked immunosorbent assay (Castelli et al., 2022), and fluorescent antibody approaches (Li J. et al., 2021; Ma et al., 2022). However, these existing methods suffer from drawbacks such as complex operation steps, the need for sample separation and purification, and limited capability to rapidly and effectively differentiate biomarker content in the sample. MNPs, on the other hand, can serve as functional materials on electrode surfaces, accelerating electron transfer, acting as carrier signal marker, biomarkers for sensitive molecular detection, or carrier signal markers themselves (Zhao et al., 2021).

Immunochromatography test strip (ICTS) hold promise for acute myocardial infarction (AMI) biomarker screening. Still, their clinical application is hindered by limited sensitivity and the absence of quantitative results. MNPs possess exceptional fluorescence quenching properties, making them suitable for marker detection applications. Gong et al. (Gong et al., 2018) devised a rapid, highly accurate, qualitative, and dual-reading ICTS that addresses the limitations of AMI biomarker screening via ICTS. The method utilized MNPs to quench the fluorescence of Cy5, which was attached to capture antibodies on test (T) lines. The sensitive quantification of cTnI and CK-MB enables the swift diagnosis of patients with urgent and severe AMI, facilitated by the fluorescence intensity shift brought about by the MNP probe. This advancement in ICTS technology overcomes previous challenges in clinical application.

**FIGURE 3:**
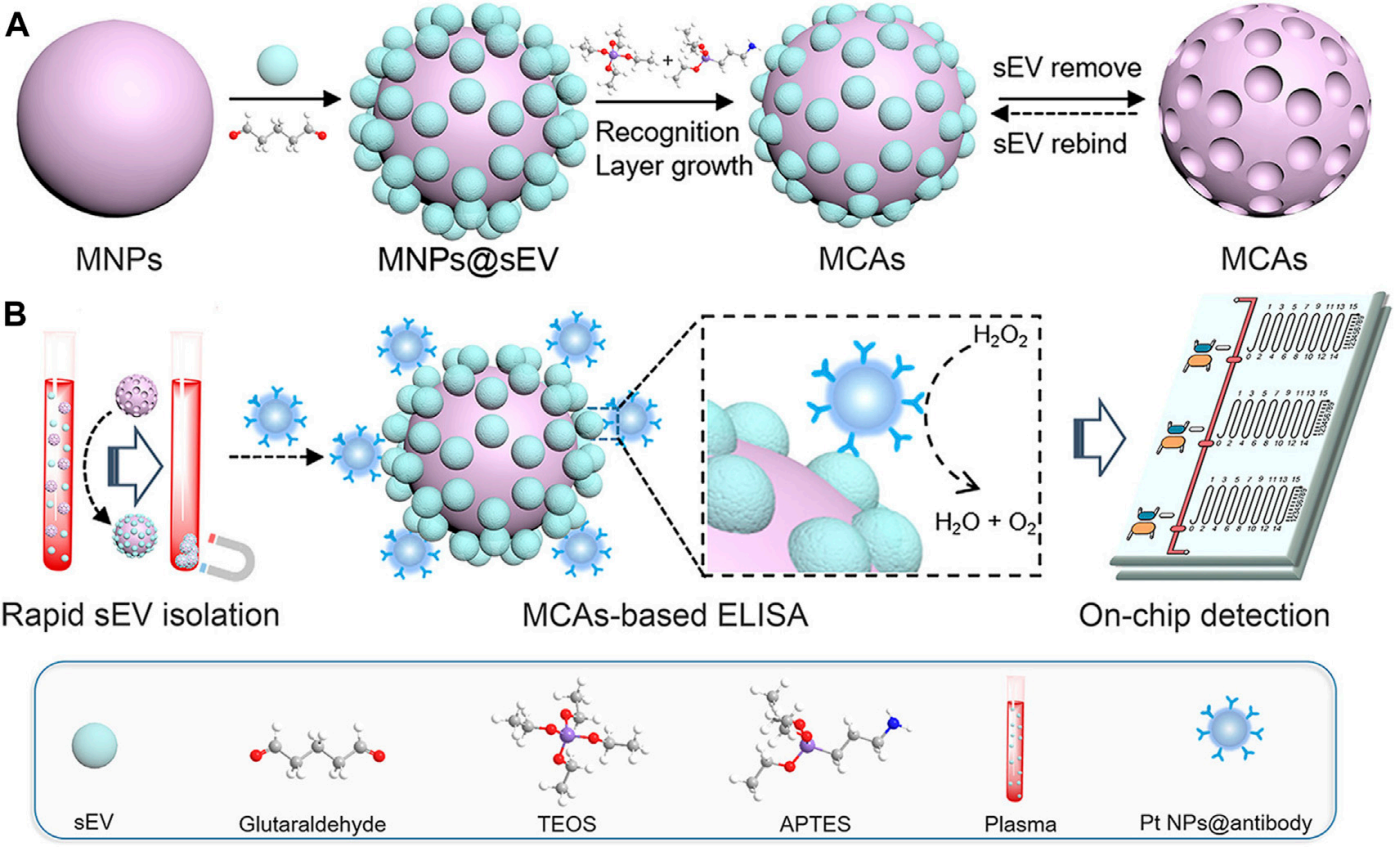
**(A)** Schematic representation of the synthetic route of MCAs. **(B)** Illustration of the MV-chip assays for small extracellular vesicle (sEV) detection. Reprinted with permission from Yang et al. (2021).

To enable magnetic capture assays (MCAs) to recognize, bind, and sense tiny extracellular vesicles (EVs) based on their form and size, Yang et al. (2021) proposed the use of MNPs (Fe_3_O_4_@SiO_2_) as carrier materials and organosilanes to create a recognition layer (Figure 3). The process involves attaching the template of small extracellular vesicles (EVs) to the MNPs, followed by culturing the SEV-modified MNPs with a mixture of organosilanes to grow the silicone recognition layer. Finally, the MNPs undergo ultrasonic treatment to remove the SEVs, resulting in three dimensional SEV imprint on the surface of the MNPs. This study introduces an advanced and innovative SEV detection platform that combines sensitivity, speed, and user friendliness, making it ideal for point-of-care testing.

The utilization of MNPs as markers and carriers of biomolecules presents a promising approach. MNPs possess unique magnetic properties, primarily owing to magnetic materials like Triiron tetroxide. Active groups are introduced on their surfaces, enabling them to bind with biomolecules such as enzymes and antibodies through coupling reactions (Popov et al., 2021). The exceptional superparamagnetic nature of MNPs allows labeled biomolecules to move directionally when subjected to an external magnetic field. Detection is performed using a superconducting quantum interference device, and a magnetic signal reader on the test paper enables rapid and quantitative detection of biological target molecules (Li Y. et al., 2021). Importantly, MNPs are biocompatible, meaning they do not induce toxicity or adverse effects in biological systems. The biocompatibility ensures their safe use *in vivo* without harm to the human body.

It is worth noting that MNPs have undergone extensive research and find wide application as biomarkers and carriers in POCT (Nasseri et al., 2018; Khizar et al., 2020; Han et al., 2021; Zhang Z. et al., 2022; Mi et al., 2022; Parihar et al., 2023a). These applications encompass immunoassays, microfluidic devices, imaging, targeted drug delivery, and more. For instance, in immunoassays, MNPs can be functionalized with specific antibodies or antigens designed to recognize target biomolecules in patient samples. This enables rapid and sensitive detection of various biomolecules, such as proteins, hormones, or viruses. MNPs also serve as biomarkers in nucleic acid amplification assay, including techniques like PCR and LAMP (Zhang C. et al., 2022). In these assays, MNPs are functionalized with specific probes that can recognize and bind to the nucleic acid sequences of interest. The interaction of MNPs with target biomolecules generates a magnetic signal that can be readily detected and quantified using a magnetic sensor or reader (Lapitan et al., 2019). This capability enables the quick and sensitive detection of nucleic acids present in blood, saliva, or urine samples from patients.

### 2.3 Rapid enrichment and purification

MNPs offer a versatile means to extract and purify molecules from complex substrates, making it easier to isolate and concentrate viral particles, proteins, nucleic acids, or other biomarkers from biological samples like blood, saliva, or urine (Chen Y. et al., 2023). This simplifies the sample preparation process, leading to quicker and improved detection. Additionally, MNPs’ ability to concentrate target analytes, reduce interferences, simplify sample preparation, and enable POC applications enhances the sensitivity, specificity, and efficiency of rapid detection methods (Eivazzadeh-Keihan et al., 2021).

Efficient isolation and enrichment of pathogenic bacteria from complex samples are vital for downstream biomedical research. Kim et al. (2021) developed a floating magnetic film using cationic polymer chains and MNPs to create a semipermeable barrier, enabling the rapid capture and enrichment in capillary glass tubes, suggesting the potential for magnetic membranes to detect large samples of bacteria and rapidly enrich biological targets in microfluidic devices. Zhou et al. (2023) introduced a method involving a composite membrane material composed of a hydrophilic membrane modified with high-density vinyl imidazole (VIM) polymer brush and Fe_3_O_4_ MNPs. This composite membrane exhibited excellent performance in antibody enrichment during purification processes.

**FIGURE 4:**
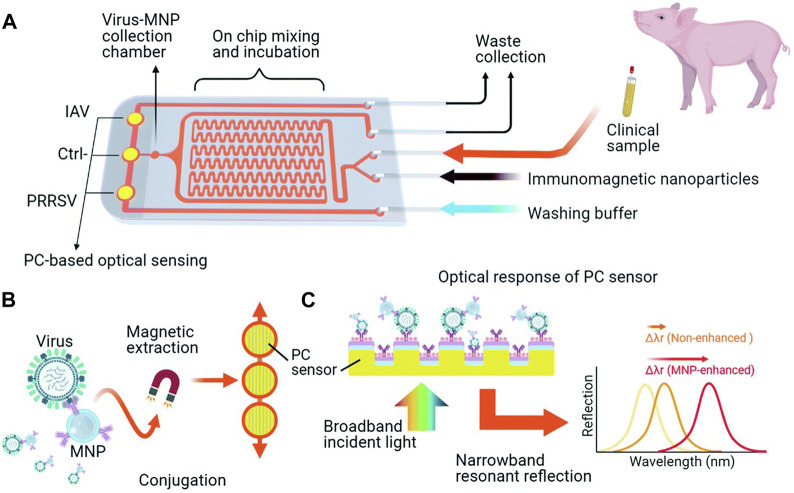
iMOF sensor chip for swine respiratory virus analysis. **(A)** Overview of the iMOF sensor chip design featuring a sample mixer, an MNP collection chamber, and three PC sensing chambers. **(B)** Integrated virus purification and detection method employing antibody-coated MNPs and PC sensors. **(C)** Diagram illustrating the label-free analysis via resonant reflection spectra measured from PC sensors, with MNPs enhancing the shift of the resonant wavelength due to the absorption of target MNP–virus conjugates. Reprinted with permission from Zhang Q. et al. (2022).

For virus detection by PCR, which typically involves samples extraction, nucleic acid purification, and detection, Zhang Q. et al. (2022) developed an immunomagnetic virus enrichment method using MNPs and a biosensor system-on-chip (IMOF) based on photonic crystals (PC) (Figure 4). This approach employed antibody-functionalized MNPs to specifically target and concentrate the virus while improving the output efficiency of the PC sensor, enabling automated detection. This technology holds promise as a rapid diagnostic tool for target virus detection.

In advanced bacteria detection technology, lateral flow assays (LFA) have gained recognition for their simplicity, low cost, short testing time, and lack of complex steps, making them important for POCT (Yu et al., 2017). Bu et al. (2020) developed a novel LFA biosensor that uses MNPs to label Amp, a beta-lactam antibiotic, facilitating the binding and enrichment of S. enteritidis in highly concentrated samples. This approach demonstrated high sensitivity and excellent selectivity for potentially interfering bacteria, enabling the quick and cost-effective real-time detection of pathogenic microorganisms.

**FIGURE 5:**
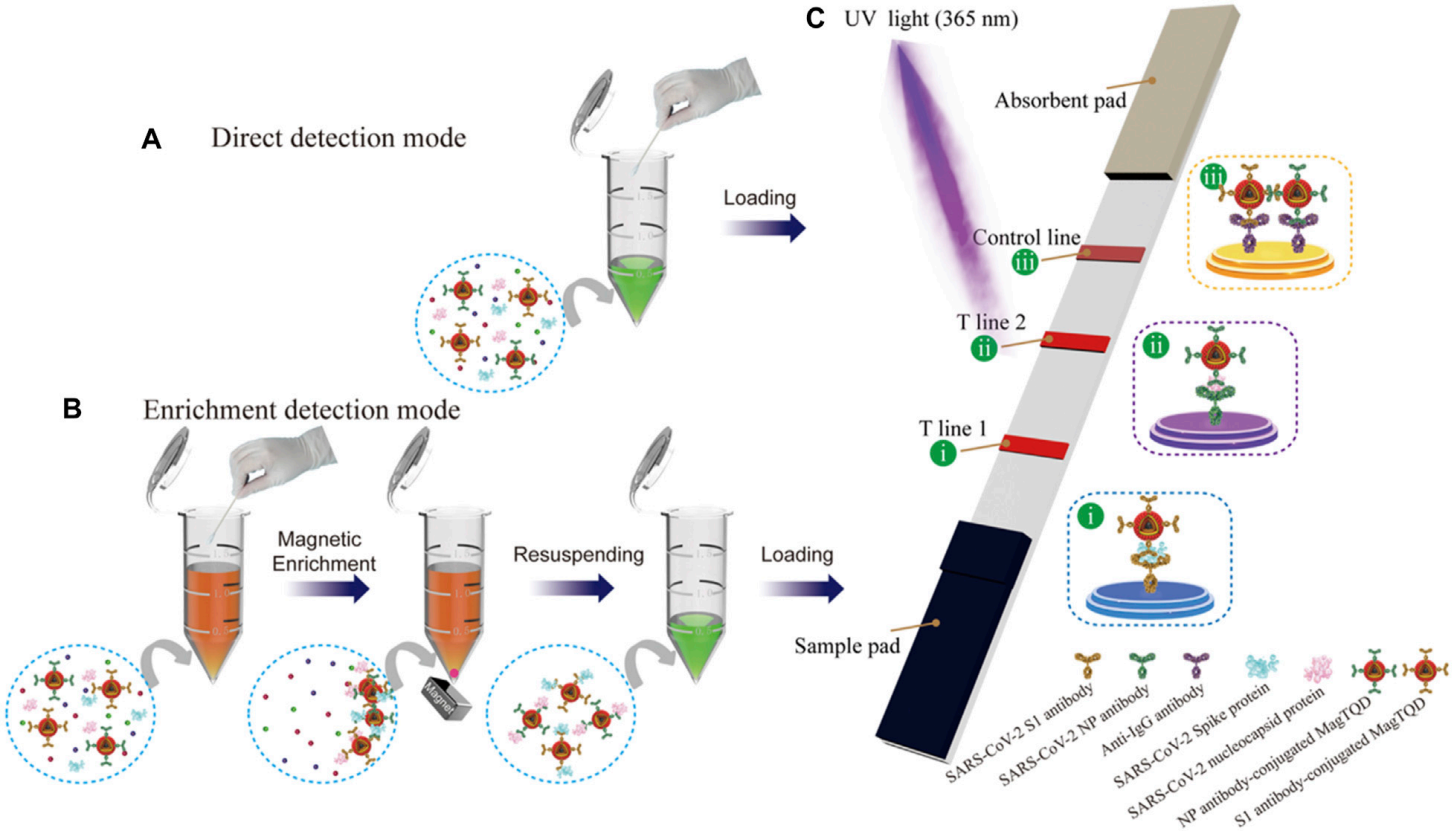
Schematic Illustration of the Dual-Mode LFIA for Simultaneous Detection of SARS-CoV-2 S and NP by MagTQD Fluorescent Tags: **(A)** Direct Detection Mode and **(B)** Enrichment Detection Mode of **(C)** Two-Channel LFIA Strip. Reprinted with permission from Wang et al. (2021).

To achieve rapid and sensitive POCT for SARS-CoV-2 infection, Wang et al. (2021) developed a fluorescent LFIA biosensor based on a bi-functional magnetic nanocomposite material. This system utilized MagTQD tags provided by Fe_3_O_4_ cores for magnetic separation and highly stable multi-layer quantum shells (Figure 5). It offered both fast direct mode for emergency screening and enriched mode for high-sensitivity quantitative analysis, with ultrasensitive detection limits for SARS-CoV-2 antigens.

Furthermore, for the purpose of enriching and identifying protein biomarkers in human plasma, Nash et al. (2010). Introduced a novel diagnostic method. This system employed pNIPAAm-MNPs capable of capturing pNIPAAmAuNPs through coaggregation, enabling efficient and thermally triggered enrichment of gold labeled biomarkers in a single step. Model studies demonstrated the system’s efficacy in purifying and enriching human plasma spiked with the model biomarker protein streptavidin. The resulting aggregates responded quickly to a magnetic field, allowing for rapid capture and enrichment.

Overall, these applications of MNPs showcase their immense potential in advancing biomedical research and diagnostic tools.

## 3 Application of MNPs-based biosensor for POCT

Target recognition stands a pivotal aspect of biosensors, necessitating the immobilization of bio-recognition molecules on MPs. Biosensors rely on various biometric mechanism, including aptamer target recognition, antibody-antigen interactions, and molecular identification using molecularly imprinted polymers (Gowri et al., 2021). Thanks to the unique properties and functions on MNPs, MNP-based biosensors have emerged as vital tools for POCT. These biosensors employ MNPs as a sensing plating platform, offering several advantages in POCT applications (Table 2).

**TABLE 2:** MNPs-based biosensor for POCT.

Biosensors type	Biosensors technology	Biosensors features	References
Magnetic Induction Biosensors	Planar Hall Effect-based magnetic sensors	High sensitivity, low power consumption, a wide operating temperature range	Hosu et al. (2019)
			Fan et al. (2021)
			Issadore et al. (2014)
	Tunnel Magnetoresistive (TMR) Sensors	High sensitivity, stability, rapid response	Denmark et al. (2019)
			Sharma et al. (2017)
			Yan et al. (2022)
	Magnetic Relaxation Switch (MRS) Sensors	High sensitivity, rapid response, tunability	Liu et al. (2018)
			Hu et al. (2022)
			Shen et al. (2022)
Electrochemical Biosensors	electrochemical impedance spectroscopy	Analyze complex systems and interfaces	Qiu et al. (2021)
			Suan Ng et al. (2022)
	electrochemiluminescence sensor	High sensitivity, low background noise, wide dynamic range	Hanoglu et al. (2022)
			Sanli et al. (2020)
	electrochemical biosensor	High specificity, rapid response, ease of miniaturization	Khoshfetrat et al. (2022)
			Chen and Wu (2022)
Optical Biosensors	Colorimetric-Based Biosensors	Ease of use, visual readouts, rapid detection	Cai et al. (2021)
			Choi et al. (2021)
			Song et al. (2011)
	Surface Plasmon Resonance (SPR) Sensors	High sensitivity, real-time monitoring capabilities, label-free detection	Sun et al. (2022)
			Liu et al. (2014)
			Bhandari et al. (2022)
	Fluorescent-Based Optical Biosensors	High sensitivity, selectivity, real-time monitoring capabilities	Xue et al. (2018)
			Wang et al. (2018)
			Chen et al. (2022b)

One of the primary advantages of MNP-based biosensors in POCT is their capability for efficient and sensitive target analyte detection. MNPs can be functionalized with specific bio-recognition target molecules, such antigens, aptamers, or enzymes, allowing them to selectively capture and detect target molecules of interest (Baig et al., 2022). This targeted recognition ensures highly specific and accurate detection, even at low concentrations, making MNP-based biosensors ideal for POCT scenarios where rapid results are imperative. Furthermore, MNP-based biosensors offer convenient and versatile signal readout options. Captured target analytes can be detected through various signal transduction mechanisms, including optical, electrochemical, or magnetic signals (Pashchenko et al., 2018). This flexibility in signal readout permits compatibility with different detection platforms, ranging from portable handheld devices to more advanced instrumentation, tailored to the specific requirements of POCT.

However, the efficient conjugation of recognition biomolecules onto MPs remains a challenge due to the inherent instability and weak affinity of bare MPs towards these molecules, hindering practical applications. Surface modification of MPs holds great promise for addressing this challenge by enhancing colloidal stability and introducing functional molecules that facilitate efficient biomolecule adherence.

### 3.1 Magnetic induction biosensors

Complex biological samples often pose challenges for conventional detection methods in the realm of biosensor technology. Magnetic biosensors have become essential for addressing these challenges, primarily due to the absence of detectable magnetic signals in crucial aspects of most biological samples. These magnetic biosensors are particularly well-suited for the development of POCT biosensors, owing to several unique properties that set them apart.

Notably, magnetic biosensors offer remarkable stability, ease of functionalization, direct signal readout, and swift separation from complex samples (Li et al., 2022). Magnetic Induction Biosensors stand out by achieving a high signal-to-background contrast while requiring minimal sample cleaning. Their ability to be customized to the size of biological targets positions them as excellent candidates for downsizing, enabling the sensitive detection of rare cells and minute molecular markers. Recent advancements in magnetic induction biosensors have showcased their notable attributes, including simplicity, reliability, cost-effectiveness, high sensitivity, and specificity. Categorized based on the versatility of MNPs, these biosensors hold the potential to revolutionize POCT diagnostics (Nabaei et al., 2018).

#### 3.1.1 Planar hall effect -based magnetic sensors

The combination of Planar hall effect (PHE) sensors and MNPs in POCT offer several notable advantages. PHE sensors, in particular, exhibit sensitivity to variations in local spin configurations and can easily derive transverse voltages, a common technique in sensor technology for imaging complex spin phenomena (Hosu et al., 2019). Additionally, PHE sensors provide a high signal-to-noise ratio and fast response time, making them valuable for real-time detection and quantification of target analytes. Consequently, this approach holds significant potential for applications in infectious disease diagnosis, cancer screening, therapeutic drug monitoring, and more.

MNPs play a critical role in effectively capturing and enriching biomolecules, thereby enhancing the assay’s detection limit. This capability enables rapid and accurate diagnosis and the POC, even when dealing with small sample sizes. By harnessing the synergistic capabilities pf PHE sensors and MNPs, the combines system exhibits improved performance characteristics, enabling sensitive and precise analysis of analytical substances in POCT settings.

The classic PHE occurs when an electrical conductor exposed to a magnetic field develops a voltage difference. This effect arises as mobile charge carriers experience deflection due to the Lorentz force, resulting in their accumulation along an aspect of the conductor (Fan et al., 2021). While PHE sensors typically exhibit lower field sensitivity compared to sensors based on magnetoresistance (MR), they demonstrate exceptional linearity even under high magnetic fields. This advantageous characteristic allows for the utilization of large magnetic fields to fully magnetize MNPs (Issadore et al., 2014). The use of MNPs and micro-Hall detectors for detecting rare cells holds immense promise for POCT diagnostics. This approach is well-suited for practical clinical environments due to its fully automated nature and minimal sample processing requirements. As an example, Issadore (2015) developed a mincrofluidic chip-based micro-Hall detector (μHD), capable of directly measuring single, immunomagnetically tagged cells in whole blood. The μHD can detect individual cells even in the presence of large number of blood cells and unbound reactants, eliminating the need for washing or purification steps. Furthermore, this cost-effective, single-cell analytical technique is suitable for miniaturization into a mobile platform for low-cost POC use.

#### 3.1.2 Tunnel magnetoresistive (TMR) sensors

A Magnetoresistive (MR) sensor is a resistor whose resistance changes when it is affected by an external magnetic field. This phenomenon, known as the magnetic resonance effect, is caused by the spin-orbit coupling between the conducting electron and the magnetosphere. The tunnel magnetoresistive (TMR) sensor is a representative of the MR sensor (Denmark et al., 2019). TMR sensors are advanced semiconductor devices that leverage the tunnel magnetoresistance phenomenon to detect variations in magnetic fields. These sensors are composed of thin layers of magnetic materials that are sandwiched between non-magnetic spacers (Lei et al., 2016). When a field of magnets is applied, the sensor’s resistance undergoes modification, enabling precise and sensitive magnetic field detection.

**FIGURE 6:**
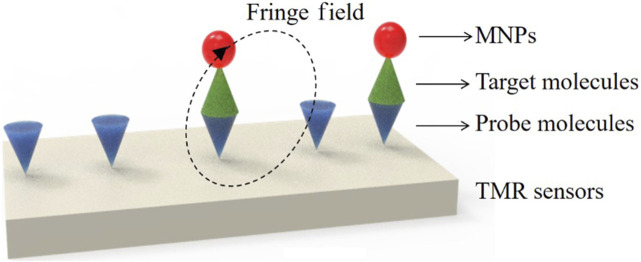
Schematics for biodetections using TMR sensors: Molecules detection. Reprinted with permission from Yan et al. (2022).

Magnetoresistive (MR) sensors, including tunnel magnetoresistive (TMR) sensors, plat a crucial role in magnetic field detection due to their unique resistance changes in response to external magnetic fields. TMR sensors, in particular, are advanced semiconductor devices that utilize the tunnel magnetoresistance phenomenon, involving thin ayers of magnetic materials sandwiched between non-magnetic spacers. When subjected to a magnetic field, TMR sensors exhibit changes in resistance, enabling precise and highly sensitive magnetic field detection. THE TMR effect, harnessed by TMR sensors, provides resistance, sensitivity, compactness, and energy efficiency, making them attractive for various biomedicals applications. These sensors are employed in determining the presence of concentration of target analyte molecules, such as DNA and proteins, often labeled with MNPs (Sharma et al., 2017). TMR sensors are invaluable tools for ultra-sensitive, multiplexed, real-time electronic sensing, thanks to their unique MR values. The fundamental operation of TMR sensors involves changing the magnetization direction of two ferromagnetic layers by applying an external magnetic field, controlling the tunnel current (resistance) perpendicular to the insulating barrier. Magnetic tunnel junctions (MTJs), the core of TMR sensors, are highly sensitive to magnetic fields and require only minimal applied magnetic fields to achieve maximum TMR. Integration of MNPs as markers enhances the signal-to-noise ratio in complex samples, making MR-based sensors particularly suitable for immediate detection in POCT. Figure 6 illustrates the detection process using TMR sensor (Yan et al., 2022). The detection process using TMR sensors typically involves pre-coating the sensor’s surface with probe molecules capable of sensing specific target analytes. These probe molecules serve as captors for the target molecules labeled with MNPs. Quantitative analysis of the signal generated allows for the determination of sample concentration. When TMR sensors are integrated with microfluidic channels, lab-on-chip systems become achievable, holding significant potential for portable POC devices.

Wu and colleagues (Wu et al., 2017) developed a rapid and highly sensitive bacterial detection method by combining a magnetic immunoassay with a TMR sensor. *Escherichia coli* O157:H7 bacteria were specifically identified by tagging them with magnetic beads through a magnetic immunoassay. The marked test strip was then subjected to the TMR for direct testing. The weak magnetic fringe field produced by the magnetic beads in an external magnetic field led to changes in the magnetoresistance of the TMR sensor. This system achieved a detection limit of 100 CFU/mL of *E. coli* O157:H7 bacteria within 5 h, showing great promise for applications in food safety and biomedical detection. It addresses the need for sensitive and efficient detection in these critical areas.

#### 3.1.3 Magnetic relaxation switch (MRS) sensors

Magnetic relaxation switch (MRS) sensors have gained significant attention in recent years for their ability to detect biological and chemical targets based on changes in the transverse relaxation time (T2) of water particles, which result from the dispersion/aggregation of MNPs (Xianyu et al., 2019). MRS sensors are highly effective because the magnetic properties of most targets are negligible, leading to minimal background interference and requiring little sample preparation. By utilizing T2 as the readout signal, MRS sensors offer nondestructive, accurate, and rapid detection of targets in complex mixtures (Liu et al., 2018).

However, one of the challenges in conventional MRS sensors has been the stability of MNPs aggregation, which relies on covalent, non-covalent, or nonspecific interactions. This has limited the sensitivity and accuracy of target detection. To address these limitations, Hu et al. (2022) proposed an advanced MRS sensor that integrated magnetic separation into the analysis process. They used MNPs of different sizes, with 1,000 nm diameter MNPs serving as the magnetic separation carrier and 30 nm diameter MNPs as magnetic signal probes. This configuration allowed for efficient separation of the larger MNPs within 0.5 min, while the smaller MNPs remained in the solution for 24 h. Compared to conventional MRS sensors, this system exhibited higher sensitivity to changes in magnetic probe concentration, resulting in improved stability and accuracy. Signal amplification strategies were also employed to enhance sensitivity, enabling the accurate and reliable detection of trace amounts of chloramphenicol.

In another example, the detection of bisphenol A (BPA) in water, a concerning environmental contaminant, was addressed using an aptamer-functionalized MRS sensor developed by Huang and Wang (2021). This sensor leveraged the high selectivity of aptamers and the excellent magnetic relaxation signal of MNPs, resulting in high sensitivity and straightforward signal readout. The aptamer-functionalized MRS sensor demonstrated great potential for practical applications in detecting BPA.

**FIGURE 7:**
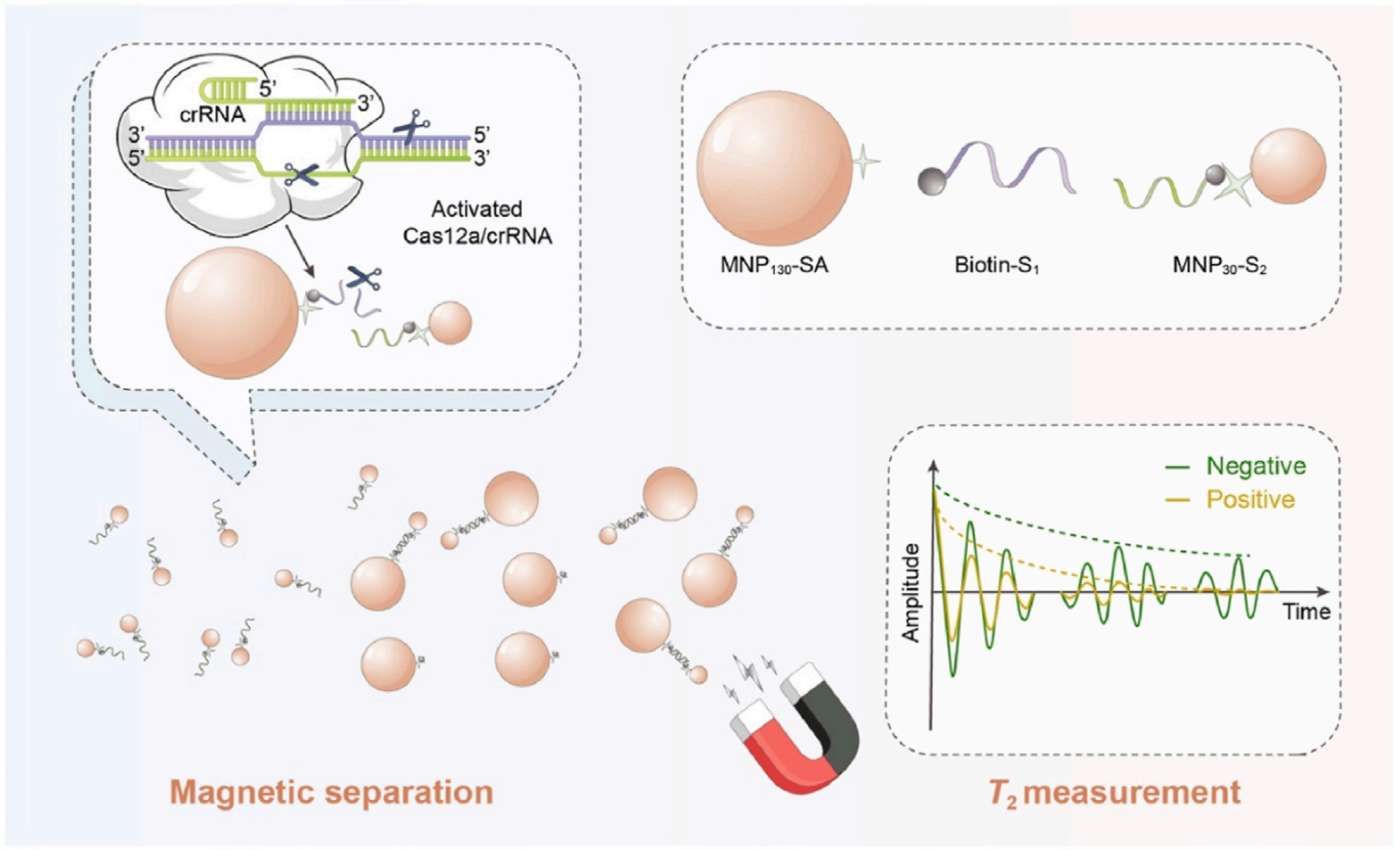
Illustration of the CRISPR-MRS biosensor for *Salmonella* detection. Reprinted with permission from Shen et al. (2022)

MRS biosensors hold promise in the field of food safety due to their simplicity and good signal-to-noise ratio. However, sensitivity and stability challenges have been encountered due to issues like insufficient crosslinking or non-specific binding of MNPs to targets. To overcome these challenges, Shen et al. (2022) integrated the CRISPR-Cas12a system into a MRS biosensor for the sensitive detection of *Salmonella*. This biosensor was designed based on the distinct magnetic properties of two sizes of MNPs (Figure 7). The presence of the target *Salmonella* triggered the collateral cleavage activity of the CRISPR-Cas12a system, inhibiting the binding of the two sizes of MNPs and resulting in an increase in unbound MNP_30_. This CRISPR-MRS biosensor demonstrated sensitive and specific detection of *Salmonella*, offering a promising alternative for pathogen detection with satisfactory sensitivity.

### 3.2 Electrochemical biosensors

The integration of MNPs into electrochemical biosensors has brought about significant advancements in POCT and biomedical diagnostics. These MNP-based biosensors combine the signal amplification capabilities of MNPs with the selective detection capabilities of electrochemicals biosensors, leading to increased sensitivity, improved selectivity, and the ability to perform multi-detection with portable diagnostic capabilities in various healthcare settings (Karimi-Maleh et al., 2020; Mollarasouli et al., 2021; Castelli et al., 2022; Mahmudiono et al., 2022; Suan Ng et al., 2022).

One key advantage of MNPs in electrochemical biosensors is their ability to amplify the electrochemical signal produced by the sensor upon binding to the target analyte. This signal amplification step significantly enhances the detection sensiticity, enabling the monitoring of low concentrations of analytes that would otherwise be challenging to detect using only the electrochemical sensor. For instance, Qiu et al. (2021) developed an electrochemical detection method that combined proximity binding-triggered hybridization chain reaction (HCR) signal amplification with the use of MNPs for efficient separation and detection of thrombin in complex sera. This method utilized magnetically separated precipitates concentrated on the electrode surface, enabling sensitive thrombin detection through amplified current signals generated by the electroactive substance MB. This approach offers high sensitivity, reduced background noise, and simple signal amplification steps, making it promising for protein biomarker detection.

MNP-based electrochemical biosensors have also shown potential in the sensitive detection of DNA methylation, which is crucial in various biomedical applications (Parihar and Khan, 2023). Khoshfetrat et al. (2022) designed a magnetic nano biosensor for DNA methylation analysis by combining it with a highly sensitive electrochemiluminescence immuno-DNA sensor. This sensor utilized a sandwiching approach, where the target methylated DNA was placed between MNPs and an anti-5-methylcytosine monoclonal antibody (MNPs/anti-5mc) and a phosphorylated DNA capture probe. This innovative approach demonstrated extraordinary sensitivity, capable of discriminating methylation levels as low as 0.1%.

**FIGURE 8:**
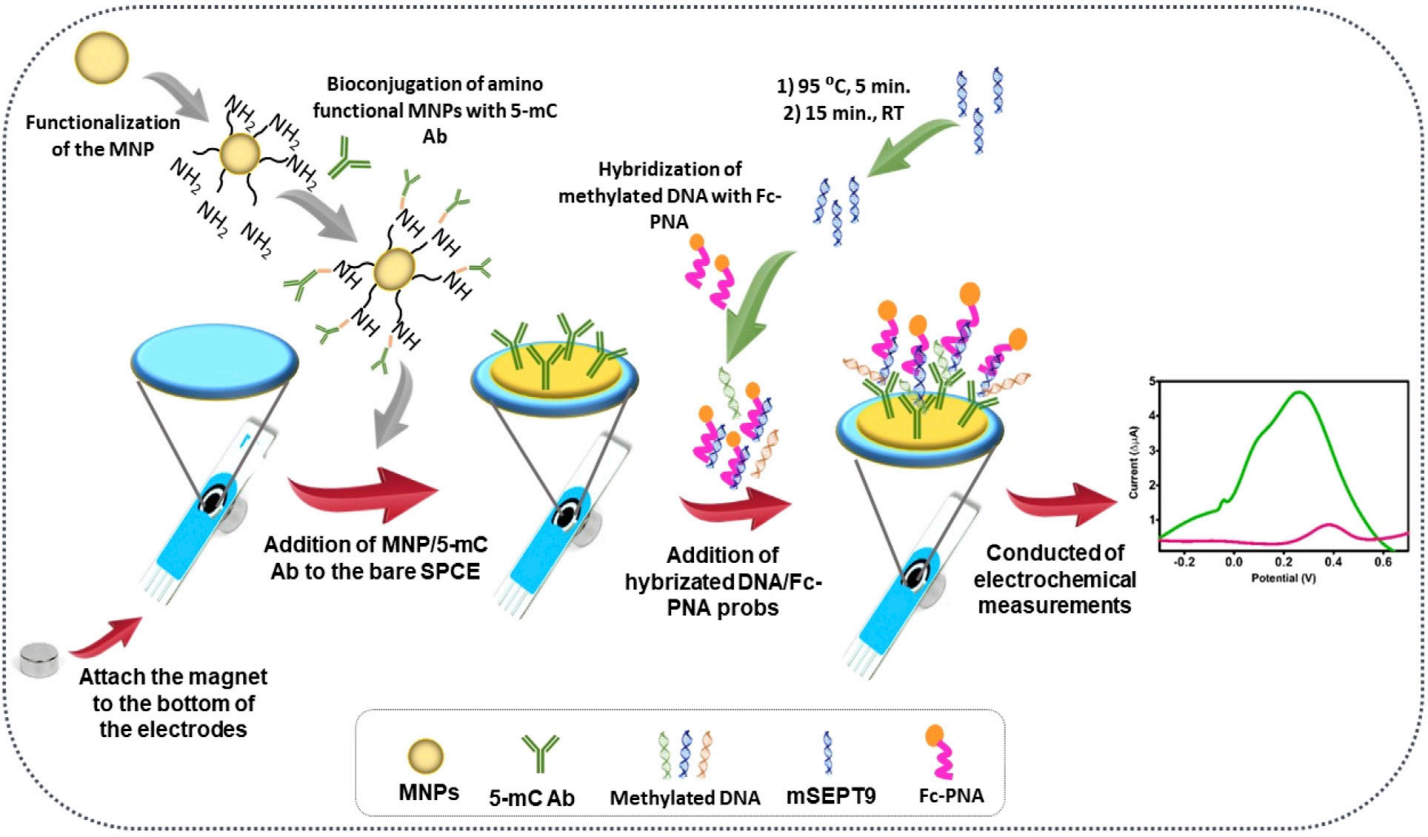
Step by step surface design and the analysis principles. Reprinted with permission from Hanoglu et al. (2022).

The incorporation of MNPs within an electrochemical biosensor leads to a significant enhancement in the performance of the sensor compared to using a solitary molecule label (Ting et al., 2009; Geagea et al., 2015). This enhancement primarily stems from the unique properties and capabilities of MNPs, resulting in a substantial amplification of the current signal. MNPs offer amplification potential comparable to that of exceptional enzyme labels in electrochemical biosensors, while also providing several advantages, including eliminating the need for timed signal recording and mitigating the risk of denaturation during storage (Cai et al., 2002). Simgle (Hanoglu et al., 2022) developed an MNPs-based electrochemical biosensor for assessing the methylation septin9 (mSEPT9) gene in early-stage colorectal cancer (CRC) (Figure 8). This biosensor leverages the stability and signal amplification capabilities of MNPs to accurately and selectively detect methylated SEPT9 gene sequences which serve as a diagnostic marker for CRC. Overall, MNPs play a crucial role in improving the performance and usability of electrochemical biosensors, making them valuable in various applications, including the early diagnosis of diseases like CRC.

The development of portable and efficient devices for POCT is crucial across various fields, including healthcare and athletics. One such application is the detection of doping substances like testosterone in sports to ensure fair competition and maintain the integrity of sports events. Sanli et al. (2020) created a portable biosensor for the detection of testosterone using a screen-printed electrode (SPE) and iron (II/III) oxide MNPs. The working electrode is the SPE, known for its simplicity and rapid response times. The proposed biosensor portability, speed and specificity make it a valuable tool for anti-doping agencies and sports organizations.

Early on, Chawla and Pundir (2011), designed a novel amperometric biosensor utilizing silicon-formed core-shell Fe_3_O_4_ (Fe_3_O_4_–SiO_2_) MNPs capable of detecting glycosylated hemoglobin effectively. The core-shell MNPs are functionalized with Ferroceneboronic acid (FAO), which is known for its ability to bind glucose molecules. FAO serves as a recognition element in the biosensor, allowing it to selectively capture glycosylated hemoglobin. This sensor is relevant in the field of diabetes management, where monitoring hemoglobin levels is crucial for assessing long-term blood glucose control.

Chen and colleagues (Chen and Wu, 2022), a fast and ultrasensitive electrochemical sensor based on magnetically trapped DNA. Thiolated MB-modified DNA probes were used to functionalize the surface of AU@MNPs. This functionalization allows the DNA probes to specifically bind to the target TP53 gene sequences. TP53 is a critical tumor suppressor gene, and mutations in this gene are associated with various cancer. The DNA probes, when attached to AU@MNPs, facilitate the concentration of target DNA sequences. This increased concentration leads to a more pronounced electrochemical signal during detection. The combination of magnetic trapping, signal amplification, and electrochemical sensing enables the rapid ultrasensitive, and specific detection of genetic mutations.

The utilization of nanomaterials, such as MNPs in electrochemical biosensors have opened up exciting possibilities for ultrasensitive and specific biological detection. One example is the detection of miR-21 with Padlock Exponential Rolling Circle Amplification (P-ERCA) and CoFe_2_O_4_ MNPs-Assisted nano electrocatalysis (Chen and Wu, 2022). The combination of P-ERCA, CoFe_2_O_4_ MNPs, and the modified graphene surface results in a significantly enhanced detection sensitivity. In addition, Pd-based nanomaterials (Pd NPs, Pd@UiO-66) are combined with catalytic hairpin assembly (CHA) to create an ultrasensitive detection of miR-211, a microRNA associated with various diseases, including cancer (Meng et al., 2020a; Meng et al., 2020b).

### 3.3 Optical biosensors

Optical biosensor systems offer an array of advantageous attributes making them highly suitable for POCT applications, particularly when combined with colorimetric and chemiluminescent detection methods. A few key benefits of optical biosensors consists of noise-free operation, inherent stability, exceptional sensitivity, simplicity and accessibility, reduced instrumentation and rapid results (Chen and Wang, 2020).

Optical biosensors showcase a diminished reliance on laboratory-specific instrumentation, making them more cost-effective and portable. With the strides made in photometry, contemporary smartphones equipped with commonplace ambient light sensors now possess the capacity to function as detectors in both systems. Colorimetric and chemiluminescent-based biosensors are user-friendly and straightforward. They often produce visible color changes or emit light, which can be easily interpreted with the naked eye. The simplicity makes them accessible to a broader range of users, including healthcare professionals with varying levels of expertise. By utilizing specific ligands or capture molecules (antibodies or aptamers), optical biosensors can achieve high specificity for target analytes. This reduces the likelihood of false results and enhances diagnostic accuracy. When combined with MNPs, optical biosensors can retain their portability. MNPs, with their small size and magnetic properties, facilitate sample manipulation and transport, making them suitable for POCT applications.

In summary, optical biosensors systems, especially those employing colorimetric and chemiluminescent detection, offer a compelling combination of sensitivity, specificity, simplicity, and portability that aligns well with the requirements of POCT. These biosensors have the potential to revolutionize healthcare by providing rapid, accurate, and accessible diagnostic solutions for a wide range of diseases and conditions.

#### 3.3.1 Colorimetric-based biosensors

In the realm of optical biosensors, the colorimetric-based biosensor constitutes a distinct category. Colorimetry, a scientific field that quantifies color, encompass various facets of photometry, including color specification, the CIE (International Commission on Illumination) system, and models for color perception and appearance (Monogarova et al., 2018). This comprehensive framework enables the quantitative representation of color in terms of intensity. Consequently, the colorimetric biosensor emerges as a sensor type that employs a comparative assessment of color intensity to quantitatively or qualitatively characterize specific analytes of interest. The presence of an analyte is often signaled by a color shift induced by chemical or light stimulation (Cai et al., 2021). This change in color results from the interaction between the chromogenic substance used as a probe and the subsequent substrate involved in a reaction with color-developing chemicals (Choi et al., 2021). Remarkably, the most notable feature of this biosensor is its ability to produce a visible color change, even discernible to the naked eye.

Colorimetric optical sensors are analytical devices designed to measure the amount of light emitted or absorbed when a bioreceptor recognizes a target molecule (Aydindogan et al., 2020). These sensors effectively convert biosensing events into observable color changes. Nanomaterials, including MNPs and AuNPs have found widespread use in this context. These systems are often characterized as simple, practical, and cost-effective, as they can be visually interpreted without the need for specialized instruments (Song et al., 2011). Among these nanomaterials, AuNPs are particularly popular in biosensor platforms due to their unique spectroscopic and colorimetric properties. AuNPs appear red when dispersed and turn blue when aggregated. This distinctive behavior has been harnessed for the detection of various biological analytes, such as cellular DNA and enzymes, making them valuable in colorimetric and UV-visible spectroscopic assays.

For instance, in Sahar’s research (Cheraghi Shahi et al., 2021), a novel colorimetric biosensor was proposed for the precise detection of aflatoxin in saffron samples. This biosensor relies on the inhibitory interaction of aflatoxin B1 (AFB1) and a bacterial enzyme digestion process. The mechanism involves impact of specific enzymatic activities on AuNPs functionalized with gelatin (AuNPs@gelatin). This approach exhibited an exceptional sensitivity, with a detection limit as low as 4 pg mL^−1^, and was successfully tested with real-world saffron samples.

Given that food is a common carrier of harmful bacteria, ensuring food quality control is of paramount importance for food safety and healthcare. Therefore, it is a critical need for the development of rapid and straightforward methods to identify hazardous microorganisms in food. Chen F. et al. (2022) This method relies on the rapid and sensitive color changes induced by the dispersion and aggregation AuNPs. The POCT visual sensing system comprises two key components: (1) an alkaline phosphatase/graphene oxide (GO@PEI-ALP) nanoconjugate that can release free ALP molecules in the presence of pathogenic bacteria; (2) D-glucose-6-phosphate (pGlu) and 3-aminobenzene boric acid (AMBA)-functionalized AuNPs (pGlu/AMBA-AuNPs) that undergo cross-linking upon pGlu digestion by free ALP molecules, resulting in significant color change. This sensing system demonstrated an impressive detection limit for target bacteria, was as low as 24 CFU mL^−1^ under optimal conditions, and proved effective for analyzing complex real-world samples.

Compared to other optical biosensing approaches, colorimetric assays exhibit tremendous potential as cost-effective and portable analytical techniques. However, further research is needed to integrate this transducer with an approach biorecognition component to develop miniature POC biosensors.

#### 3.3.2 Surface plasmon resonance (SPR) sensors

Surface plasmon resonance (SPR) biosensors have become indispensable tools for POCT of across various biomarker classes due to their user-friendly operation, rapid response times, and exceptional selectivity (Sun Y. et al., 2022). SPR is a powerful and widely adopted technology in biological and chemical sensing, facilitating real-time monitoring of molecular interactions in POCT diagnostics (Chiu, 2022). MNPs have gained recent attention for their integration into SPR systems for biomolecule immobilization and purification. MNPs are incorporated into SPR for several reasons: first, they possess a large surface area, allowing for a high density of biomolecule immobilization; second, their strong magnetism enables the direct capture, separation, and concentration of target molecules through an external magnetic field; and third, their high refractive index and molecular weight effectively enhance SPR signals. These characteristics endow MNPs with the dual function of amplifying SPR sensor sensitivity and acting as concentration purifiers to eliminate background interference from unknown molecules in SPR experiments.

Detecting small molecules or trace analytes directly using conventional SPR sensors can be challenging due to the minimal changes in refractive index resulting their binding to the sensor surface. Liu et al. (2014) introduced an innovative method for deltamethrin detection by integrating SPR sensor technology with Fe_3_O_4_ MNP tests. The carboxyl groups on Fe_3_O_4_ MNPs surfaces facilitate antibody functionalization. Fe_3_O_4_ MNPs, coupled with antibodies, serve as “carriers” rapid transfer of target analytes from a sample to the sensor surface and as labels to enhance SPR detection sensitivity, given their high refractive index and molecular weight. Consequently, Fe_3_O_4_ MNP-anti-deltamethrin monoclonal antibody conjugates allow for direct detection of deltamethrin on the chitosan-modified SPR sensor surface. This method offers accurate and sensitive deltamethrin detection and can be adapted to detect other analytes of interest by modifying the appropriate antibody in the MNP conjugates.

*Salmonella* currently represents one of the most prevalent foodborne bacteria, responsible for numerous illnesses, hospitalizations, and fatalities worldwide. While various technologies are available, SPR has emerged as an advantageous option due to its real-time detection capability coupled with high sensitivity and specificity. In Bhandari’s research (Bhandari et al., 2022), a rapid and accurate methods for detecting *Salmonella typhimurium* is presented, utilizing SPR biosensors combined with antibody-coupled MNPs amplification. The study employs two monoclonal antibodies specific to flagellin, with one attached to MNPs and the other immobilized on the sensor surface. A sensor surface bearing these antibodies reacts to flagellin and MNPs. The results demonstrate that SPR biosensors, when combined with MNPs, can significantly improve the accuracy and sensitivity of detecting various pathogens and biomolecules.

#### 3.3.3 Fluorescent-based optical biosensors

Fluorescence stands as one of the most commonly employed methods in the realm of optical biosensors, widely utilized for its ability to detect alterations in the fluorescence properties of a molecular recognition element when it interacts with a target molecule.

**FIGURE 9:**
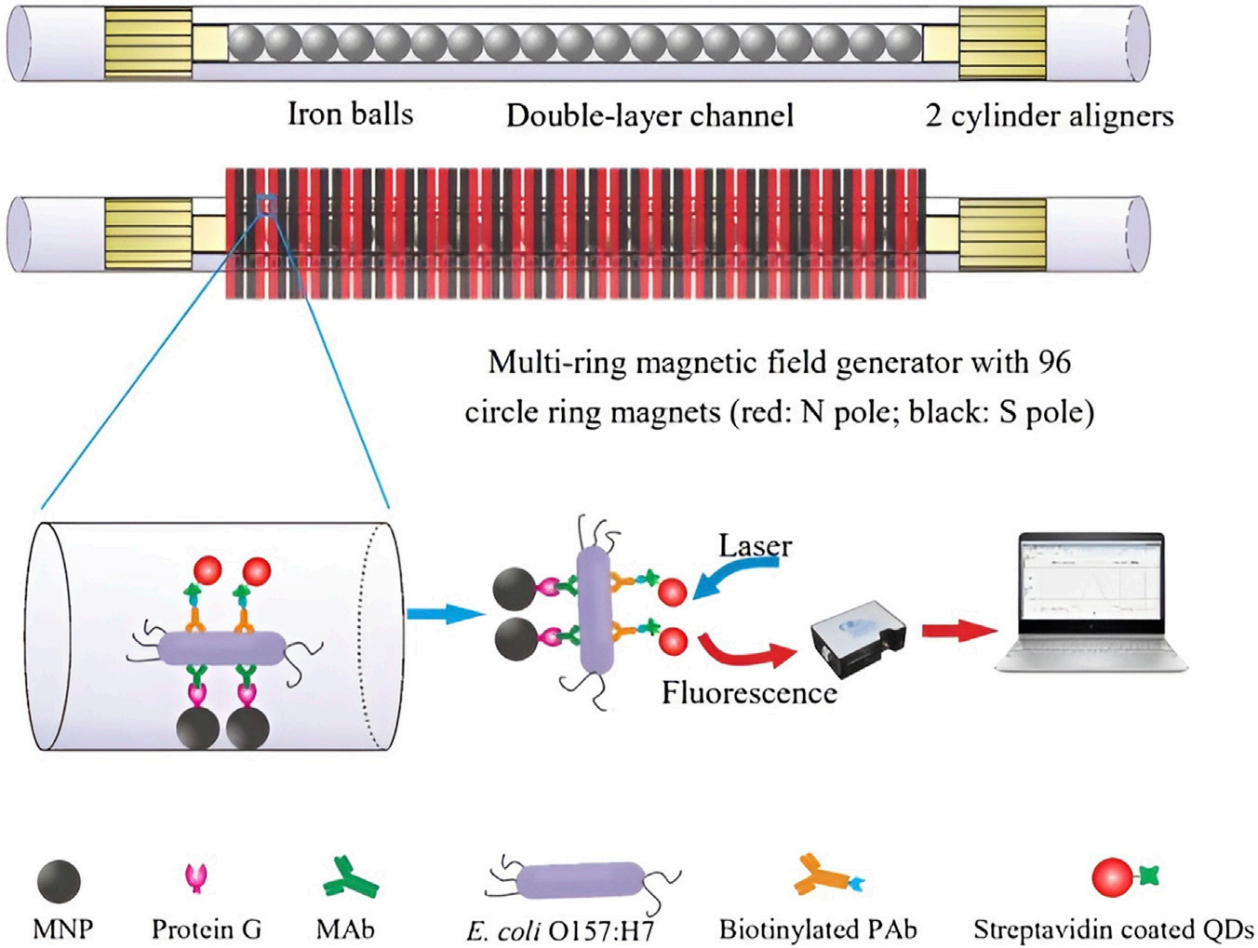
Schematic of the ultrasensitive fluorescent biosensor using double-layer channel with MNPs and quantum dots for rapid detection of foodborne pathogenic bacteria.Reprinted with permission from Xue et al. (2018)

For example, in the context of rapidly screening contaminated food products, Xue et al. (2018) developed an innovative fluorescent biosensor utilizing a double-layer channel with immune MNPs to efficiently separate and concentrate target bacteria (Figure 9). The biosensor also incorporated immune quantum dots (QDs) and a portable optical system to quantitatively detect *E. coli* O157:H7 cells in the sample. The fluorescence intensity measured by the portable optical system facilitated the identification of the target bacteria. Moreover, this biosensor can be adapted for the detection of various foodborne pathogens and biological targets by modifying the antibodies, demonstrating potential for multiplexed and high-throughput detection.

In another study, Wang et al. (2018) designed an environmentally friendly and sensitive OTC fluorescence sensor. This sensor utilized carbon dots (CDs) as a fluorescent probe to detect OTC in the presence of Fe_3_O_4_ MNPs and H_2_O_2_. Fe_3_O_4_ MNPs acted as catalysts for peroxidase-like reactions. Additionally, the absorption band of OTC overlapped with the excitation bands of CDs, resulting in the quenching of CD fluorescence by OTC. The sensor’s feasibility for OTC detection in drugs was further confirmed, highlighting its simplicity, sensitivity, and high selectivity. Compared to existing detection methods, this approach offers advantages such as simplicity, cost-effectiveness, and rapid detection, making it a practical tool for OTC detection, food safety analysis, and clinical diagnosis.

Recently, Chen et al. (2022b) developed a fluorescence biosensor based on magnetic Fe_3_O_4_-modified graphene oxide (MNPs@GO) for rapid and direct diagnosis of *S. aureus*. The biosensor employed aptamer-functionalized upconversion nanoparticles (UCNPs) in combination with MNPs@GO. In the presence of *S. aureus*, MNPs@GO underwent cleavage, and GO was not magnetically separated, resulting in a decrease in upconversion fluorescence (UCF) intensity at 547 nm from aptamer-functionalized UCNPs to GO due to fluorescence resonance energy transfer (FRET). The detection of *S. aureus* in chicken samples illustrated the potential of this method for bacterial safety monitoring in food.

## 4 Concluding insights and future prospects

This comprehensive review highlights the significant role of MNP-derived biosensors in the field of POCT. The review explores various biosensing technologies enabled by MNPs, including magnetic sensing biosensors, electrochemical biosensors, and optical biosensors, all of which demonstrate great potential for the development of POCT devices. Optical biosensors coupled with MNPs are particularly emphasized due to their advantages, such as low background signal, cost-effectiveness, stability, and ease of functionalization.

Biosensors play a crucial role in manufacturing of POCT devices, and recent research has witnessed rapid advancements in the development of smart biosensors. MNPs offer distinct advantages in this context, including low background signals in biological samples, affordability, stability, and ease of functionalization. The integration of MNPs with optical biosensors and smartphones holds promise for the development of portable, accessible, and digitized POCT devices that can seamlessly integrate with medical databases.

Moreover, certain biosensors offer multiplex measurement capabilities, enabling the extraction of valuable biological information from small sample volumes, including whole blood. The trend toward instrument miniaturization and portability ensures that POCT devices can cater to diverse scenarios and meet the specific requirements of various healthcare settings. Although many advances have been made in the field of POCT testing, there are still some challenges in the manufacturing of rapid testing equipment. First and foremost, maintaining device performance and integrating complex detection technology under the premise of miniaturization is a key issue. Secondly, ensuring accurate results in clinical use requires POCT equipment to have a high degree of stability and reliability. Finally, striking a balance between reducing device costs and improving performance is an important challenge. Addressing these challenges necessitates interdisciplinary cooperation, and we look forward to continued efforts and innovation in the field of POCT equipment manufacturing to make greater contributions to human health.

In conclusion, MNP-derived biosensors are poised to revolutionize POCT devices. Their adaptability, sensitivity, and multifaceted applications make them indispensable tools for enhancing diagnostic capabilities at the POC. The integration of optical biosensors with MNPs, along with the progress in smartphone-based platforms, paves the way for portable, accessible, and digitally-integrated POCT devices. As we continue to harness the potential of these biosensors, along with the power of information storage databases and artificial intelligence algorithms, we can look forward to advanced and personalized healthcare solutions that will significantly enhance patient outcomes in the future.
